# Rights-Based Priorities for Children with SEND in the Post-COVID-19 Era: A Multi-Method, Multi-Phased, Multi-Stakeholder Consensus Approach

**DOI:** 10.3390/children12070827

**Published:** 2025-06-23

**Authors:** Emma Ashworth, Lucy Bray, Amel Alghrani, Seamus Byrne, Joanna Kirkby

**Affiliations:** 1 School of Psychology, Liverpool John Moores University, Liverpool L3 3AF, UK; j.kirkby@ljmu.ac.uk; 2 Faculty of Health and Social Care, Edge Hill University, Ormskirk L39 4QP, UK; brayl@edgehill.ac.uk; 3 School of Law and Social Justice, University of Liverpool, Liverpool L69 7ZR, UK; a.alghrani@liverpool.ac.uk; 4 Manchester Law School, Manchester Metropolitan University, Manchester M15 6BX, UK; s.byrne@mmu.ac.uk

**Keywords:** special educational needs and disabilities, COVID-19, children’s rights, education, health and social care

## Abstract

**Background:** The provision of education, health, and social care for children with special educational needs and disabilities (SEND) in England has long been criticised for its inequities and chronic underfunding. These systemic issues were further exacerbated by the onset of the COVID-19 pandemic and the accompanying restrictions, which disrupted essential services and resulted in widespread unmet needs and infringements on the rights of many children with SEND. This study aimed to use a three-phase consensus-building approach with 1353 participants across five stakeholder groups to collaboratively develop evidence-informed priorities for policy and practice. The priorities sought to help address the longstanding disparities and respond to the intensified challenges brought about by the pandemic. **Methods:** A total of 55 children with SEND (aged 5–16), 893 parents/carers, and 307 professionals working in SEND-related services participated in the first phase through online surveys. This was followed by semi-structured interviews with four children and young people, ten parents/carers, and 15 professionals, allowing for deeper exploration of lived experiences and priorities. The data were analysed, synthesised, and structured into five overarching areas of priority. These were subsequently discussed and refined in a series of activity-based group workshops involving 20 children with SEND, 11 parents/carers, and 38 professionals. **Results and Conclusions:** The consensus-building process led to the identification of key priorities for both pandemic response and longer-term recovery, highlighting the responsibilities of central Government and statutory services to consider and meet the needs of children with SEND. These priorities are framed within a children’s rights context and considered against the rights and duties set out in the United Nations Convention on the Rights of the Child (1989). Priorities include protecting and promoting children with SEND’s rights to (1) play, socialise, and be part of a community, (2) receive support for their social and emotional wellbeing and mental health, (3) feel safe, belong, and learn in school, (4) “access health and social care services and therapies”, and (5) receive support for their parents/carers and families. Together, they highlight the urgent need for structural reform to ensure that children with SEND receive the support they are entitled to—not only in times of crisis but as a matter of routine practice and policy.

## 1. Introduction

Among the most vulnerable children and young people in our society are those with special educational needs and disabilities (SEND) [1]. SEND is a term within an English context which reflects the fact that children with a special educational need (SEN) may also possess a disability, and therefore fall within the legal ambit of the additional protections afforded by the Equality Act 2010 [2]. Under section 20(1) of the Children and Families Act 2014 (CFA) [3], a child or young person “has special educational needs if he or she has a learning difficulty or disability which calls for special educational provision to be made for him or her”, while section 20(2) proceeds to state that such a difficulty or disability means they have “a significantly greater difficulty in learning than the majority of others of the same age” or that the disability “prevents or hinders him or her from making use of facilities of a kind generally provided for others of the same age in mainstream schools or mainstream post-16 institutions”. Children and young people with SEND are entitled to provision to be made for them in school through ‘SEN Support’. If a child or young person with SEND requires more provision than is available through SEN support, an Education, Health and Care Plan (EHCP) can be obtained through the process of an Education, Health and Care Needs assessment. The purpose of an EHCP, which should be holistic and person-centred [4,5], is “to meet the special educational needs of the child or young person, to secure the best possible outcomes for them across education, health and social care and, as they get older, prepare them for adulthood” [6] (p. 142).

In comparison to their peers without SEND, children with SEND are more likely to qualify for free school meals [7] and be exposed to poverty [8]. Education, health, and social care provision for children with SEND in the UK has experienced chronic underfunding by an estimated £1.5bn [9,10], and has been described as severely flawed and inequitable [11,12,13]. Furthermore, the process for obtaining SEND support has been characterised by “confusion and at times unlawful practice, bureaucratic nightmares, buck-passing, and a lack of accountability, strained resources and adversarial experiences” [14] (p. 3).

The emergence of SARS-CoV-2, leading to COVID-19, exacerbated many of these difficulties. The declaration of COVID-19 as a global pandemic by the World Health Organisation in March 2020 prompted a series of measures by the UK Government to curb transmission, including directives such as working from home (where possible), the closure of certain businesses and venues, restrictions on public gatherings, and advice on handwashing and respiratory hygiene [15]. Redeployment of healthcare staff to COVID-19 related duties also took place and schools closed to all pupils except those of key workers and children categorised as vulnerable. One of the first legislative changes enacted under the Coronavirus Act 2020 concerned the formal diminution of the legal standard contained in section 42 of the CFA (2014), which places an ‘absolute duty’ on Local Authorities (LAs) to meet the education and health care needs of children and young people with SEND. This was replaced by a ‘reasonable endeavours’ duty, which in England was enacted without the benefit of a children’s rights impact assessment. From 6th January 2021 until 29th March 2021, a second national lockdown occurred, during which time children and young people were again mandated to engage in learning and education from home whenever possible. During the lockdowns, children and young people with an EHCP were considered ‘vulnerable’ by the UK Government and should have been able to continue attending school in-person, whereas children in receipt of SEN support only were not. Therefore, a significant number of children with SEND (1.1 million) in receipt of SEN support did not have the opportunity to continue attending school in-person [16].

Evidence indicates that the education of children with SEND was adversely affected by the pandemic [1,13,17]. For instance, the ‘Ask Listen Act’ Study [18] found that 69% (n = 509) of parents/carers thought that the national lockdowns had a negative impact on their child’s education and learning, and 89% (n = 655) reported that their child was not able to access face-to-face education during the pandemic. Likewise, other studies show that between March 2020 and February 2021, of 3487 families surveyed, only 30% of children with SEND continued attending school [19] and only 6% of children with EHCPs went to school between March and May 2020 [20]. Moreover, remote learning was reported as not working well for most children with SEND [11,21], with parents noting that it was not effective in meeting their child’s needs [18]. For many children, there was no individualised support, work was not differentiated, online lessons were not adapted, and minimum adjustments were denied [17,21,22].

Health and social care support for children with SEND and their families was also hugely affected and reported as insufficient, with many essential appointments and assessments being delayed, rescheduled, or cancelled [18,19,21,23,24]. In 2021, 64% of parents reported receiving a decreased level of health and social care support (including from educational psychologists [EPs], occupational therapists [OTs], speech and language therapists [SALTs], and Child and Adolescent Mental Health Services [CAMHS]) for their child and, as a result, over half of parents (68%) stated that their child’s physical health declined [25]. Further studies suggested that many children with SEND’s mental health and wellbeing also deteriorated during the pandemic [10,13,18,19,21].

However, despite existing evidence pointing towards a disproportionate negative impact of the pandemic on children and young people with SEND, there has been a noticeable lack of solution-focused research exploring strategies and priorities to support this demographic in the post-pandemic landscape. This paper outlines the collaborative process of identifying priorities for policy and practice, aimed at ameliorating the enduring consequences of the COVID-19 pandemic on children with SEND, employing a rights-based approach. This multi-phase, multi-stakeholder consensus-building study consisted of mixed-method online surveys (Phase 1), semi-structured qualitative interviews (Phase 2), and activity-based group workshops (Phase 3), dedicated to establishing priorities for children with SEND, encompassing those receiving both SEN support as well as those with an EHCP.

## 2. Methods

### 2.1. Design

This cross-sectional, multi-phase, mixed-methods study aimed to rapidly map overlapping priorities from different perspectives and identify mutual priorities across stakeholder groups. The rapid approach was appropriate given this study was conducted during the pandemic and the findings were needed to inform decision-making. An iterative consensus-building process utilised a range of methods in order to give all stakeholder groups, particularly children with SEND and their parents/carers, a voice [26], at what was a time of national and global restriction. This approach aligned in some regards with a modified Delphi approach [27], with Phase 1 collecting a detailed dataset of priorities across stakeholder groups which were then expanded, refined and condensed through iterative, flexible consultation. We based our approach on group decision-making and consensus as defined by the World Health Organisation [28], where consensus was interpreted to mean “general acceptance” by those involved. Our decision-making processes throughout the project were transparent and collaboratively made alongside the stakeholder groups. We felt strongly that highly prescriptive Delphi approaches often exclude children, particularly children with SEND, and instead frequently base consensus development and decision making with ‘expert’ adult proxies, instead of adapting approaches to acknowledge that children are experts on their own lives in matters relating to them [29]. Thus, the consensus-building approach used here was designed to be inclusive, empowering all those involved, especially children and young people, to gain general agreement on the key priority areas. This is aligned with existing work in the field using multi-phase consensus-building methods with children with disabilities and parents/carers [30,31].

During Phases 1 and 2, perspectives and insights were sought through surveys and interviews with children with SEND, parents/carers, and professionals regarding the COVID-19 pandemic and the associated restrictions, and included specific questions designed to elicit participants’ foremost concerns regarding policy and practice for children with SEND during pandemic management and in the post-pandemic era. Responses were then presented in activity-based group workshops (Phase 3), which were instrumental in the collaborative development of the identified priorities. Informed, opt-in consent (and child assent) was sought from all participants, with accessible and ‘easy read’ information sheets provided. The study was funded by the National Institute for Health Research (NIHR202718) and ethical approval was provided by Liverpool John Moores University’s Research Ethics Committee (21/PSY/030 and 21/PSY/024).

### 2.2. Public Involvement and Engagement

Our team engaged in extensive consultations with children and parents to inform the design of the study, reported according to the GRIPP-2 short form [32]. Four young people from an online youth forum advised on the questions, proposed format, and suitability of the surveys and interviews for children. Additionally, we consulted further with three children with SEND via remote conversations. These consultations resulted in changes to the study information sheets, survey questions, and response options. For the parent/carer survey, we sought guidance via telephone consultations from two parents of children with SEND. Their insights helped to refine the information sheets, recruitment methods, materials, and interview questions. For Phase 3, we collaborated closely with a group of nine children and young people with SEND (aged 8–15 years) from a local youth centre. Through a face-to-face meeting, this group offered valuable input on the structure and content of our workshops, advising us to create separate activity stations within a room, allowing children to move between activities and freely exchange their perspectives, with the ‘time talking’ by the adults kept to a minimum. Professionals from a range of disciplines working within SEND services formed the steering committee for the study, providing guidance over the course of the project.

### 2.3. Data Collection and Analysis

**Figure 1:**
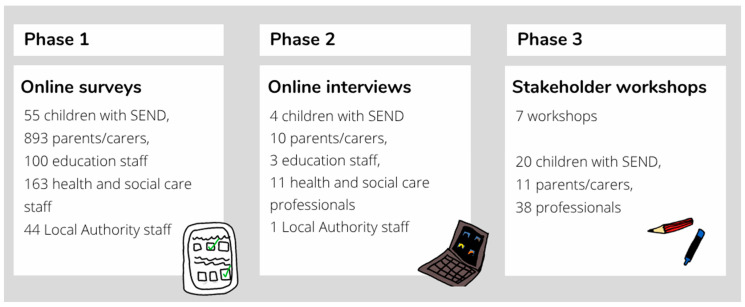
Overview of methods used in this study.

An overview of the data collection methods across the multiple phases are presented in Figure 1.

#### 2.3.1. Phase 1: Mixed-Method Online Surveys

Mixed-method online surveys were administered to children with SEND, parents of children with SEND, health and social care professionals, education professionals, and Local Authority staff. Participants were recruited via opportunity and snowball sampling, using flyers posted on social media and through the distribution of study information via key organisations working with children with SEND and their families. Inclusion criteria were broad—any children or young people aged 5–16 years with any form of SEND (diagnosed or awaiting diagnosis, SEN support or EHCP), parents/carers of children aged 5–16 years with any form of SEND, and any professional working across education, health or social care, or within Local Authority services, with children or young people with any form of SEND.

The children’s surveys contained a suite of different response options, to foreground individuals’ needs and abilities (including drawing and uploading pictures, typing responses, and selecting emojis). At the end of the online survey, children with SEND were asked: ‘if you were in charge of the country, what would you do to help children with special educational needs and disabilities over the next year?’. Parents/carers were asked to identify their top priority for their child over the next year, and professionals were asked to identify their top three priorities for (1) funding and (2) policy over the next year. The survey data were collected between June and August 2021. Responses were anonymous.

Following data collection, the ‘priority question’ data for each participant group were collated. A conventional content analysis approach [33] was used to inductively code and categorise the data, whereby the text responses from each participant group were coded and then similar priorities grouped together. Team members EA, LB and AA each analysed the data from one professional group, while JK analysed all data from young people and parents/carers. Any uncertainties about codes were discussed at regular team meetings. A tally system was then used to log the number of times each coded priority was reported by each participant group. Based on the priorities that received the most tallies, the priorities were further refined to identify the most frequent or ‘top’ priorities from each participant group. These priorities were presented at two steering group meetings, which identified the need to develop an integrated set of priorities across all participant groups, as well as for the priorities to more clearly delineate pandemic-related challenges from historical issues with the SEND system (see Supplementary Materials Figure S1 for detail). This discussion also highlighted that there was an overlap and repetition between the responses from professionals on funding and policy priorities, and so it was decided to merge these priorities. Once merged, the priorities were grouped into five key areas. Qualitative interview data (phase 2) were then analysed separately before being mapped onto the existing priorities identified from the surveys (see below).

#### 2.3.2. Phase 2: Semi-Structured Qualitative Interviews

Participants were asked to self-identify at the end of the online survey if they were interested in taking part in an online semi-structured qualitative interview. Interviews were conducted on MS Teams between August and September 2021, using questions and prompts to elicit more in-depth information from participants. Adjustments were made to interviews where needed to ensure children and young people could take part, and we provided opportunities for children and parents to be interviewed separately. Activity books were sent to children before their interviews so that they could prepare, which included information about questions that would be asked and places for them to note down ideas they wanted to share. The researcher also offered short ‘say hello’ meetings with children prior to the interview, to help ascertain communication preferences and styles, and any technology needed to support the interview. Interviews were then adapted accordingly.

At the end of the interview, parents were asked to identify priorities for moving forward e.g., ‘what do you think is important for you and your child as we move forward from the pandemic? (prompt-education, health care, social care, play/recreation/social skills/friendships)’, and professionals were asked ‘what do you think is important for the SEND children you work with as we move forward from the pandemic? (prompt-education, health care, social care, play/recreation/social skills/friendships)’, while children and young people were asked ‘if there was another lockdown what should be done to help children with SEND?’.

The interview data were analysed according to reflexive thematic analysis [34]. Multiple team members (EA, LB, JK, AA) began by inductively coding the interview data, with each team member analysing data from one stakeholder group. Codes were then reviewed by a second team member and collated into potential themes in an iterative process, returning to the data and relabeling codes to develop the themes. Potential themes were shared with the team for feedback, and the developing themes were further refined and defined. Finally, the data relating to the priority questions were mapped against the five priority areas identified from the survey data, allowing for the identification of new priorities and areas of overlap. The priorities were then updated accordingly, ready to be presented and discussed at the workshops (see below). Analysis was collaborative, and we acknowledge the multiple world (academics, parents, carers) and disciplinary (health, psychology, law) views of the researchers involved, which may have shaped interpretation. In line with a reflexive approach, we did not aim for ‘accurate’ or ‘reliable’ coding [35] or the use of rigid coding frameworks when analysing the qualitative data. When discussing findings, the team reflected on the assumptions and expectations they brought to the work, as well as how their own experiences may have influenced their interpretations of the data.

#### 2.3.3. Phase 3: Group Priority-Setting Activity-Based Workshops

Participants were recruited via opportunity/volunteer sampling. Children and young people were recruited to take part in one of three face-to-face workshops via two specialist provision schools and a national charity supporting children with SEND. Parents/carers were recruited to take part in one of two face-to-face workshops via a parent/carer support forum and a national charity supporting children with SEND and their families. Professionals were recruited via flyers posted on social media to take part in one of three online workshops.

Children, young people, and parents/carers in the face-to-face workshops were given the opportunity to either write, use post-it notes, draw, or be supported by a scribe to share their views onto a large sheet of paper, which outlined the five priority areas developed from survey and interview data from the previous phases. The second activity involved drawing, writing, or dictating views onto ‘thumbs up’ and ‘thumbs down’ templates , to illustrate things that they thought had worked well, and not worked so well, during the pandemic. In the final activity, children and young people were provided with blank postcards and a post box, where they could write, draw, or dictate a message to the Prime Minister, sharing what they felt was the ‘most important’ thing that needed to be done to help children and young people with SEND recover from the pandemic (see results section for illustrations of the data collection tools). Children were supported to engage in the workshops in the way that suited them best (e.g., writing, drawing, a scribe). As workshops were conducted in schools or through organisations that already supported the young people, their communication needs (e.g., sign language) were facilitated by the school staff, parents/carers, and charity workers who usually supported them in the setting. Children could engage in all the activities or choose to only share their views using one activity. We provided a quiet space in each room, if needed by children, young people or parent/carers. Field notes of discussions were taken, and we also took photos of the large sheets of papers and other written/drawn elements. The in-person workshops with children with SEND and parents/carers took place between October and December 2021.

During the online professionals’ workshops, the evidence from the surveys and interviews was shared in the form of a pictorial representation of the five priority areas (see Supplementary Materials Figure S1), to solicit professionals’ views on each of the areas, what each area might look like in practice, and to talk about anything they felt was missing or required refinement. The online workshops were audio-recorded. They took place online between September and November 2021.

Workshop data were analysed using content analysis procedures [33], as described above, in an iterative process with the workshop participants. The children, young people, parents/carers, and professionals were an integral part of the analysis process and decision-making, as part of the workshops involved helping the research team sort and organise the priorities within the key priority areas. The priority areas’ labels and contents changed based on participants’ views and input. We did not conduct any analysis specifically on the drawn images, as we had accompanying text or notes taken of verbal explanations from the children and young people; this felt important to prevent us misinterpreting any images based on our worldviews and adult perspectives.

## 3. Results

### 3.1. Participants

In total, 893 parents/carers, 55 children with SEND, and 307 professionals (163 health and social care, 100 education, 44 Local Authority) completed the survey. Children were aged 5–16 (mean = 11.3 years) and 93% (n = 48) were White British or Irish. Overall, 55% (n = 29) identified as male and 40% (n = 21) identified as female; the remainder chose not to say. They were predominantly based across England, although a minority of participants were also located in Scotland (6%; n = 3) and Wales (1%; n = 2). In terms of support needs, 60% (n = 32) had a communication and interaction need (e.g., autism, 57% (n = 30) had a cognition and learning need (e.g., learning difficulties), 42% (n = 22) had social, emotional and mental health difficulties (e.g., anxiety disorder) and 23% (n = 12) had sensory and/or physical needs (e.g., cystic fibrosis). Regarding parents/carers, 88% (n = 799) were White British or Irish. The majority (96%; n = 848) identified themselves as female, 4% (n = 37) were male, and 0.2% (n = 2) chose ‘not to say’. In terms of their children, 67% (n = 600) reported that their child had communication and interaction needs, 52% (n = 465) had cognition and learning needs, 42% (n = 379) had social, emotional and mental health difficulties, and 34% (n = 306) had sensory and/or physical needs (parents could tick as many boxes as applied). Regarding schooling, 58% (n = 519) of parents/carers reported that their children attended mainstream school, 25% (n = 224) were in a special school, 1% (n = 8) were in a pupil referral unit or alternative provision, 4% (n = 33) were home educated or flexi-schooling, 3% (n = 22) were in a private or independent school, and 0.1% (n = 1) were in a residential school. Finally, professionals were predominantly based across England, although a small number of participants were located in Wales (1%; n = 4). Professionals’ job roles included social care (8%; n = 24) or SEND-specific social care (7%; n = 23), community primary care (17%; n = 53) or SEND-specific primary care (11%; n = 33), school teacher (9%; n = 28), teaching assistants (7%; n = 22), school leadership (9%; n = 28), and school SEND coordinators (SENDCos) (14%; n = 44).

Ten parents/carers, four children and young people, and 15 professionals participated in semi-structured interviews. Children aged 8–14, all male, with SEND including autism, ADHD, sensory differences, specific learning difficulties (SpLDs) and mental health needs took part in an interview. Parents were all female, and had children with SEND including autism, ADHD, sensory needs, mental health needs, genetic conditions and SpLDs. The professionals’ job roles included Educational Psychologist, school SEND coordinators (primary and secondary), Deputy Headteacher in a special school, Family and Specialist Support Service Manager, Community Physiotherapist, Designated Clinical Officer, Therapy Manager, and Local Authority Youth Voice Lead.

In total, 11 parent/carers, 20 children with SEND, and 38 professionals participated in the activity-based group workshops. No demographic information was formally collected, although children and young people with a range of support needs were involved, including ADHD, autism, learning disabilities, sensory impairments, and physical disabilities. Professionals’ job roles included Head of SEND, SEND advisor, and SEND consultant at local councils, paediatrician, advanced nurse practitioner, learning disability specialist in CAMHS, early years worker, and children’s holiday club worker.

**Figure 2:**
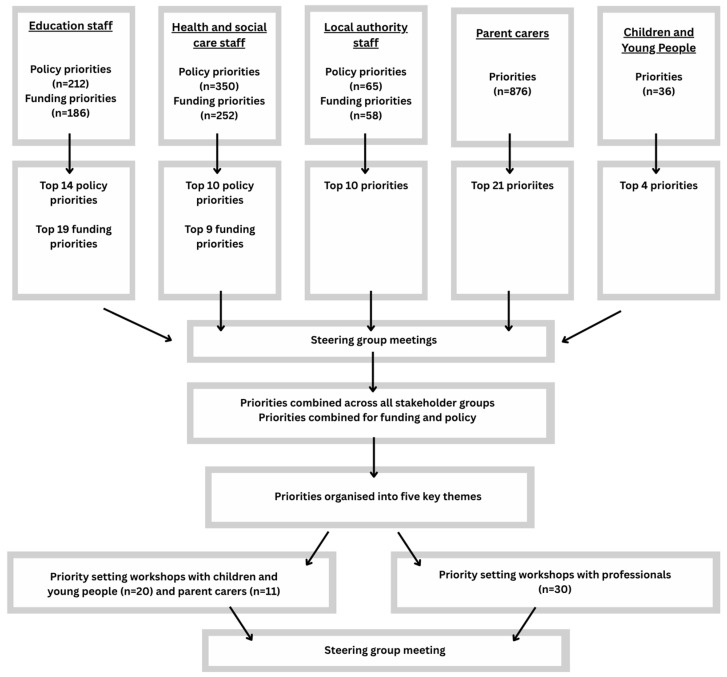
Date prioritsation processes.

We will firstly present the priorities identified by each participant group from the surveys and interviews, and then describe how these were refined and consensus was reached within the workshops. Figure 2 outlines the processes undertaken at each Phase.

### 3.2. Surveys and Interviews

36 priorities were identified from children and young people’s (n = 55) survey responses, which were grouped into three areas of education, friends, and mental health. 876 priorities were identified from the parents/carers’ (n = 893) surveys; these were tallied before being collated into the ‘top twenty’ priorities. For the education professionals, 212 policy priorities and 186 funding priorities were identified (n = 100); these were collated and tallied into the top 14 policy priorities, and 24 top funding priorities (many had an equal number of tallies). Health and social care professionals (n = 163) identified 350 policy priorities and 252 funding priorities across the survey responses; these were analysed and refined into the top ten policy priorities, and top nine funding priorities. Local Authority staff (n = 44) identified 147 policy priorities and 27 specific funding priorities; these were refined and analysed to the ‘top ten’ policy and funding priorities (as there was significant overlap between them). Interview data were then mapped onto the relevant priorities, to provide further context and rationale for each, whilst remaining open for any priorities not previously identified to be added. No new priorities were identified from the interview data alone. The priorities for each stakeholder group are presented in Table 1 and Table 2.

### 3.3. Workshops

Prior to the workshops, the priority areas for each stakeholder group (Table 1 and Table 2) were synthesised to remove duplicates, and merged and grouped together to encompass priorities from all the stakeholder groups. These broad priority areas were organised into five key areas: (1) opportunities to socialize and have fun, (2) support for social, emotional and mental health, (3) flexibility, choice and support in school, (4) access to services and therapies to stay healthy, and (5) support for parents/carers and family (see Supplementary Materials Figure S1 for an overview). Priorities within each of the areas were then presented in turn at each workshop, asking participants to sort, refine and organise the priorities, and to identify if any priorities were missing.

### 3.4. Children’s Activity-Based Group Workshops

**Figure 3:**
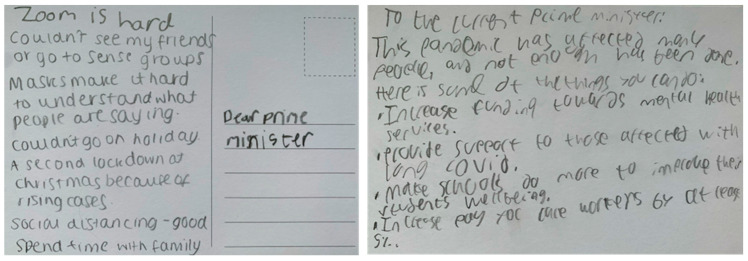
Messages to the Prime Minister from young people with SEND.

**Figure 4:**
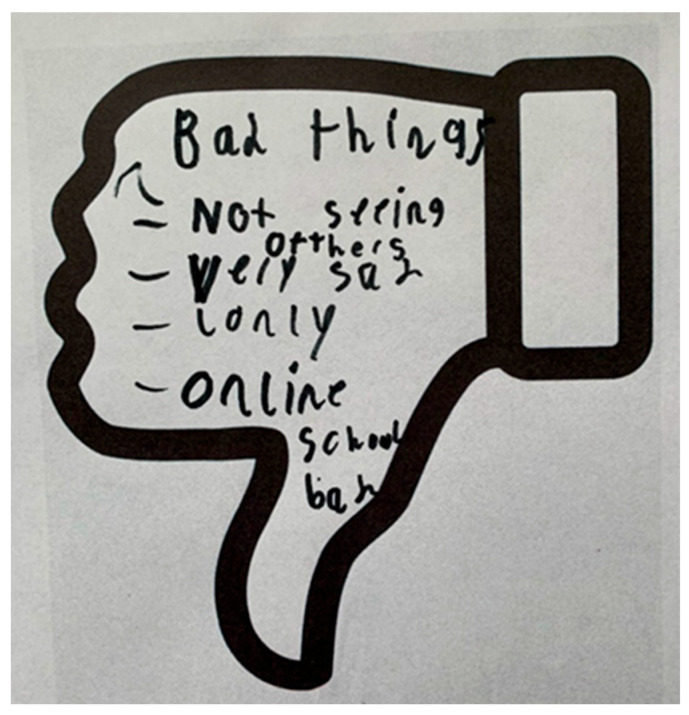
Bad things about the pandemic and restrictions from a child with SEND.

The children in the workshops discussed the priority areas from their perspectives and identified additional priorities within these. Many of the children told us that their mental health had deteriorated during the lockdowns, resulting in them feeling sad, lonely, and anxious. To counter this, children and young people suggested that increased funding for mental health services was a priority following the pandemic. Additionally, they explained that they would like the school environment to be one where they feel they can ‘belong’, which helps them want to attend school and enables them to ‘*feel safe in school*’. The children who continued to attend school in-person over the lockdowns explained that they had a better experience at school during these times, as there was more one-to-one help to complete schoolwork and ‘*school was quieter and better*’. In terms of lessons, children would like more variation, ‘*to learn skills to get a job*’ and for ‘*lessons to be more fun*’, for example, being able to go swimming, do more physical education, and play more games. Outside of school, children would like to have ‘*places to have fun and be with friends*’, make new friends, and participate in activities. The pictures in Figure 3 and Figure 4 provide examples of what children told us through the workshop activities.

### 3.5. Parents/Carers’ Activity-Based Group Workshops

Many of the comments in the workshops related to ‘*everything being reactive not proactive; only giving help once in crisis—only once a family is in breakdown*’ and how the SEND system forced parents ‘*to focus on what is wrong with my child rather than their strengths*’. Parents/carers told us that they felt the pandemic had a detrimental effect on their child’s mental health, and that more mental health provision is needed for children with SEND, so that they can discuss their feelings and receive support, to prevent a mental health crisis from occurring. Parents/carers felt that in order for this to be effective, mental health professionals need a better understanding of SEND and offer alternatives to talking therapies, including ‘*more fun options such as walks, nature or games*’. It was also identified that it can take longer for children with social, emotional and mental health (SEMH) needs to build trust with professionals and ‘*by the time the child starts opening up in counselling then the block is over*’. In terms of education and learning, the transition back to school was overwhelming for many children with SEND, and parents/carers felt that more time and space to catch up socially would have been helpful for their children when returning to school. Parents/carers also felt it would have been beneficial for schools to provide their children with more opportunities to develop their independence and life skills, and pursue their special interests, rather than exclusively focusing on catching up with the curriculum. Additionally, parents/carers felt it was important for mainstream schools to have more SEND trained staff, and for them be more inclusive.

With regard to health and social care, while some parents/carers did note that professionals delivered excellent care during the lockdowns, going ‘*above and beyond*’, most parents/carers said that health and social care and respite provision was unavailable. Parents/carers said that they would have welcomed a regular ‘check in’ phone call from professionals during this time. However, parents/carers also said that while phone consultations and online meetings were helpful for some children with SEND, they were difficult for others, and, as such, suggested that children could be given the option of a face-to-face or online sessions for future appointments. Parents/carers also highlighted the lengthy delays and many obstacles they faced when trying to access specialists to obtain diagnoses, treatment, or therapy. Parents/carers prioritised the need to reduce waiting lists and the lack of support along the journey; ‘*you have to fight for anything*’, and also afterwards; ‘*you get the diagnosis and don’t get offered anything*’.

Parents/carers explained how they felt overwhelmed and exhausted having to ‘*fight for any kind of support*’ for their child and navigate the SEND system. Many parents discussed how advocates to guide families through the process of obtaining support were invaluable but often non-existent; ‘*you need someone who gives advice on options, where to go, who to speak to for support and help with forms’.* They also discussed the survey and interview findings which highlighted a lack of local places for children with SEND to socialise, have fun without judgement, and feel part of the community. These elements were highlighted as a ‘lifeline’ for both parents/carers and their children, but often were described as oversubscribed; ‘*they shouldn’t have to wait 2 years to go to the cinema*’.

### 3.6. Professionals’ Group Workshops

The workshops highlighted examples where professionals had worked ‘over and above’ their role during the pandemic to navigate around restrictions and deliver services to children with SEND and their families. However, professionals also reported that the entire workforce (across education, health, social care, and government) needed improved trained on SEND-related issues as ‘*teachers, TAs, senior leaders, do not have enough experience and knowledge to put enough intervention and support in for children with SEND*’, and particularly around the link between mental health difficulties and disability to ‘*make sure no diagnostic overshadowing is going on*’. They spoke about how a graduated response to mental health support was needed, from specialists who can support a child in crisis to lower-intensity wellbeing support in school. They also felt as a whole the education system needed changing to prioritise and promote children’s mental health; ‘*I do not feel that the education system as it is now is fit for purpose, I don’t think it’s helpful to any young person’s mental health*’. Professionals commented that following the pandemic, schools should focus on the wellbeing of children rather than ‘catching up’ on education and learning. According to professionals, remote learning worked well for a minority of children with SEND due to a lack of inclusivity in the school setting and therefore felt that ‘*schools should also consider that there should continue to be an online offer because a lot of our young people thrived in lockdown because they didn’t have to be in that environment*’. Conversely, they also spoke about how some children with SEND were home-schooled ‘*not because it was chosen, but because they couldn’t cope going to school*’, thus highlighting that schools need to become more inclusive. Furthermore, professionals felt that community inclusion was important for children with SEND, and strategies for this should be integrated into EHCPs.

Professionals highlighted how they saw parents/carers who were exhausted and ‘*stressed out*’ during the pandemic, as social care support, such as respite services, ceased, and emphasised a need for ‘*holiday activity settings with specialised provisions that can cater for mixed abilities and accessible provision*’. They also commented on a significant increase in requests for SEND support, which they could not meet due to a lack of funding and resources. As a result, professionals felt that existing challenges with service delivery had been amplified, but their services remained understaffed as no new staff were coming entering the workforce; ‘*we have a recruitment and retention problem and heads reporting that they are unable to recruit to positions*’. As a result, professionals suggested that greater opportunities to train more healthcare workers (especially speech and language therapists) were needed.

### 3.7. Priorities for Policy and Practice for Children with SEND

Developed through iterative consultation and collaborative decision-making, the priorities identified from Phases 1 and 2 were organised and synthesised into five key areas, before being further refined during Phase 3. The full set of priorities for policy and practice, including responsible organisations and links to children’s rights (in accordance with the United Nations (UN) Convention on the Rights of the Child (CRC; [36]) are included in Supplementary Materials Table S1. However, Table 3 provides a summary of key priorities.

The priorities were grounded in a child-centred approach and aligned with the rights of children as outlined in the UN CRC [36]. They are applicable to all children and young people aged 5–16 with SEND. Whilst these priorities for policy and practice have been framed by the rights of the child as recognised under the CRC, those working with children and young people with SEND need to also recognise the legal entitlements which they further possess under both the Equality Act 2010 [2] and the CFA 2014 [3]. Whilst this project and the developed priorities aimed to be solution-focused and forward thinking, it is important to recognise that they are positioned within a SEND system which is acknowledged as underfunded and typically poorly equipped to meet the children with SEND’s needs. In order for these priorities to be realised and for children with SEND’s rights to be met, increased and sustained investment is needed from the Government across all sectors, as well as the proper implementation of the current SEND legal framework. The project also identified, as is well evidenced in existing literature, that there should be more integrated working across all services and between all professionals who work with and support children with SEND, as well as improved accountability and clearer lines of responsibility, to ensure equitable access to support across all regions of the UK.

## 4. Discussion

This paper outlines the process of conducting a multi-phase, multi-stakeholder mixed-methods study to collaboratively develop priorities for policy and practice from different perspectives, to ameliorate the enduring consequences of the COVID-19 pandemic on children with SEND. By employing a rights-based approach, we identified five key priority areas highlighted by parents/carers, children and young people with SEND, and professionals: *(1) My right to play, socialise, have fun, and be part of my community; (2) My right to support for my social and emotional wellbeing (SEW) and mental health; (3) My right to flexibility, choice, and support so I can feel safe, belong, and learn in school; (4) My right to health and social care services and therapies in order for me to stay healthy*; and *(5) My right to support for my parents/carers and my family*. We endeavoured to create and develop these rights-based priorities with those most impacted, namely children with SEND and their parents/carers. In positioning these priorities against the duties which the UK Government have assumed pursuant to the CRC, which includes, amongst other rights, the right to rest, play and leisure (Article 31 CRC), the right to the highest attainable standard of health (Article 24 CRC), respect for the views of the child (Article 12 CRC), and the right to family support and an adequate standard of living (Article 27 CRC), the succeeding section engages with the wider children’s rights aspects which the priorities identified within this study relate to.

Another policy prioritisation study for child public health during COVID-19 [37] described the ‘collateral damage’ (p. 533) to young people caused by the unintended consequences of COVID-19 restrictions in England. The priorities they identified were broadly similar to those identified here, including ensuring delivery of healthcare, mitigating the impact of disrupted schooling, supporting children’s deteriorating emotional wellbeing and mental health, and addressing child poverty and social inequalities. However, they noted how children and young people with SEND faced additional pressures during this period. What has become clear from the burgeoning literature on the impact of COVID-19 and associated restrictions on children and young people with SEND is the insufficient attention which the English Government accorded to their needs and rights [13]. This in turn caused harm and exacerbated already prevalent inequalities for this vulnerable cohort [18]. The legal measures enacted in the Coronavirus Act 2020 in England were affected within a rights-based vacuum and were bereft of a children’s rights or equality rights impact assessment. The consequences of these omissions were that the specific rights-based considerations were inconsistent with the guidance issued by the UN Committee on the Rights of the Child, the treaty-monitoring body of the CRC, who advocated for governments to consider, amongst other matters, the “health, social, educational, economic and recreational impacts of the pandemic on the rights of the child” [38] (p. 1).

This assumes increased significance given that pre-pandemic SEND provision was already in a state of flux and decline. Research commissioned by the Department for Education and conducted by Adams et al. [39] identified several key sources of parental dissatisfaction with the EHCP process. These included poor communication from local authorities, a lack of accessible information and support throughout the process, limited transparency around delays, insufficient involvement of families, inadequate detail within the EHCPs themselves, and a general failure to foster effective collaboration with schools and other agencies. Further highlighting systemic weaknesses, a 2019 Parliamentary report described the SEND framework as being plagued by confusion, unlawful practices, excessive bureaucracy, lack of accountability, insufficient resourcing, and an overly adversarial experience for families [14]. Similarly, OFSTED [40] reported that as of August 2019, 50 of the 100 Local Authority inspections completed (out of a total of 151) had resulted in a requirement to produce a Written Statement of Action due to significant weaknesses in their SEND arrangements. More broadly, research by Robinson et al. [41] raised concerns that the implementation of the new legal and policy frameworks governing SEND provision coincided with a period of national and local austerity, leading to inconsistent and often “patchy provision” for children and young people. While acknowledging the positive intent behind such reforms, they cautioned that without robust oversight, such changes risked falling short of their goals, and recommended that the Government establish nationally consistent quality assurance and accountability mechanisms, underpinned by clear local structures and a well-trained specialist workforce, to ensure equitable and effective provision for all children with SEND, including those with EHCPs. Additionally, a 2018 survey conducted by the National Association of Head Teachers (NAHT) [42] found that 94% of respondents reported increased difficulty in securing the necessary resources to support pupils with SEND compared to two years earlier. Notably, 73% attributed these challenges to broader financial cutbacks across the education sector.

Similarly, in their evaluation of the SEND framework two years post-implementation, Tysoe et al. [43] found that SENDCos—key figures in the coordination and delivery of educational provision—were frequently unable to carry out their roles effectively. This was due to a combination of systemic issues, including increased administrative demands, poor communication from Local Authorities, insufficient external service provision, financial pressures, and delays in completing statutory needs assessments. These interconnected challenges significantly hindered the overall effectiveness of service delivery. Such conclusions corroborated earlier findings by Boesley and Crane [44] who highlighted the concerns of SENDCos that the prevailing focus on academic attainment continued to shape perceptions of EHCPs primarily as educational tools, rather than as the integrated, wraparound support documents originally envisioned in the SEND reforms. Therefore, taken together, the pre-pandemic evidence on the reform and delivery of SEND services paints a fragmented and disjointed picture, revealing a system in which service provision was often inconsistent and difficult to navigate. Such shortcomings not only adversely affected children, their rights, and their long-term development and wellbeing, but also significantly undermined the core objectives of the Children and Families Act 2014.

Whilst the present study has confirmed the disproportionately negative impact of the COVID-19 pandemic on children and young people with SEND, what is now required is the robust articulation of the needs and priorities of children and young people with SEND as we enter the pandemic recovery phase. Central to these aims moving forward is the need for children’s rights law, as per the rights contained within the CRC, to be centrally considered within any future pandemic management. As Byrne and Lundy [45] (p. 358) have previously reminded us in pre-pandemic times, “most public policies that affect children and young people, whether directly or indirectly, do not reference the CRC; indeed, many will have been designed by officials who have limited or no knowledge of its existence”. Such observations clearly materialised during COVID-19 when children’s rights law was inadequately given effect to, or complied with, during the legislative and policy responses to the pandemic. As Byrne and Lundy [45] further argue, a child rights-based approach to policy making and delivery should involve the adoption of the CRC as the legal foundation upon which such policies are based on, and further that the process should directly involve children and young people, “and build their capacity as rights-holders to claim their rights” (p. 358). Indeed, Byrne and Lundy [46] (p. 274), have elsewhere noted that the obligations arising under the CRC are “largely confined to the margins of policy-making”.

However, it is arguably within the context of a global pandemic that the rights and needs of the most vulnerable, and in particular children with SEND, should have been prioritised. The evidence from this research not only underlines the disproportionally adverse effects which COVID-19 had on children with SEND, but also exposes the pervasive rights-infringing impact which it exerted. From education, access to emotional and mental health support, to the ability to engage in play and recreation and maintain friendship groups, children and young people with SEND within this study have been clear on where future priorities must lie in the event of prospective lockdowns, pandemics, or restrictions. In this regard, it is contended that the rights contained within the CRC must become the legal bedrock of any future pandemic or lockdown management, with full consideration given by all policymakers to how the rights of children, and especially the most vulnerable, can be given full legal effect.

At the level of implementation, the primary responsibility for enforcing not only the specific priorities identified in this study, but the broader spectrum of children’s rights in the event of future lockdowns or public health restrictions, rests with central Government. This is principally due to its exclusive legislative authority to introduce and enforce such emergency measures. As this study contends, the protection and promotion of children’s rights must be foregrounded in any future legislative or policy response to emergent crises. This necessity is further underlined by findings from the global *CovidUnder19* initiative, which captured the perspectives of over 26,000 children across 137 countries concerning the recognition of their rights during the initial six months of the COVID-19 pandemic. The study reported that children widely perceived their voices as marginalised, concluding that “their governments were not considering children as a priority and were definitely not seeking their views when crucial policy responses to the pandemic were formulated and implemented” [47] (p. 281). Significantly, the study emphasised that “at times of crisis, children’s rights … are not a dispensable luxury but an indispensable entitlement” [47] (p. 282). These findings underscore the critical need for child rights respecting governance and the systematic incorporation of children’s rights into emergency response frameworks moving forward.

Children’s rights must become operationally centralised within all decision-making structures to avoid them becoming peripheral considerations or overlooked afterthoughts [47]. This includes ensuring that fundamental children’s rights principles such as non-discrimination (Article 2 CRC), the best interests principle (Article 3 CRC), the right to life, survival and development of the child (Article 6 CRC), and the right of the child to participate in matter affecting them (Article 12 CRC), are properly assimilated into all decision-making structures at both macro and meso levels. Children’s rights law also necessitates that governments, in all their manifestations, including the responsibilities which fall on Local Authorities, “undertake all appropriate legislative, administrative, and other measures for the implementation of the rights recognised in the present Convention” (Article 4 CRC). This fundamental obligation cuts across the entire implementation of the CRC and has been interpreted broadly by the UN Committee [48]. It requires, amongst other matters, reviewing of existing domestic legislation, visible cross-sectoral coordination between all levels of Government and between Government and civil society, the adoption of comprehensive and cohesive rights-based national strategies which are embedded in the convention, training and awareness raising, and the development and expansion of effective policies, programmes and services which establish real and achievable targets that transcend abstract statements of policy and practice. For children and young people with SEND, the above requirements impose clear and ongoing obligations on the state to ensure that the rights and needs of such children are upheld. In practical terms, and as we now enter the recovery phase of COVID-19, it means that central Government, in collaboration with Local Authorities, should partake in an ongoing review, to make sure that sufficient staffing, resources (technical, financial, informational etc.), and facilities are provided to ensure that the needs of children and young people with SEND are met. More widely, it also means that in the event of any future lockdowns or restrictions which impact children and young people with SEND, that the rights-based framework as contained within the CRC becomes the basis upon which decisions affecting children with SEND are instituted upon.

The suggestions outlined above assume increased importance in light of recent findings by the Children’s Commissioner for England [49], who noted that children with SEND were not only more likely to feel less safe and lonelier than their non-SEND peers, but further, that their educational, health, and social care needs were disproportionately unfulfilled. This included long delays in the identification of their needs, the recognition that schools were often unable to meet their needs, the inaccessible nature of many children’s services including playgrounds, toilets, and wider leisure activities, poor quality care, discrimination, and the disruptive nature of the transition between important services for children and young people with SEND. Such findings, which corroborate many of the conclusions from the stakeholders within this paper, strengthen the call for a more cohesive, coordinated, and rights-based approach to the planning and provision of services for children with SEND.

### Limitations and Future Directions

The present study has several limitations to consider. Firstly, the individuals who participated in this study were self-selecting and so may not be representative of the wider population. Indeed, the most isolated parents/carers and young people may not have had the time or technological resources to take part in a predominantly online study. Furthermore, while we made significant efforts to ensure that all children and young people who wanted to take part were able to do so, it is wholly possible that not all children’s needs were accounted for. In addition, participants in the final phase (workshops) were not anonymous to one another, which may have introduced social desirability bias. Further research should seek to conduct longitudinal research to assess whether and how identified priorities are taken up by the Government, and what outcomes they produce. Comparative research across national or regional contexts would also help elucidate context-specific barriers and enablers to inclusive policy implementation.

## 5. Conclusions

The consensus-building process utilised in this study sought to be inclusive, empowering all those involved, especially children and young people, to gain agreement on key priority areas. The priorities for both pandemic response and longer-term recovery highlight the responsibilities of central Government and statutory services to consider and meet the needs of children with SEND, in order to protect and promote children with SEND’s rights to (1) play, socialise, and be part of a community, (2) receive support for their social and emotional wellbeing and mental health, (3) feel safe, belong, and learn in school, (4) “access health and social care services and therapies”, and (5) receive support for their parents/carers and families. Together, the priorities highlight the urgent need for structural reform to ensure that children with SEND receive the support they are entitled to—not only in times of crisis but as a matter of routine practice and policy.

## Figures and Tables

**Table 1:** Most frequent priorities for children and young people with SEND after the COVID-19 pandemic identified by children and parents in the surveys and interviews.

**Children and Young People’s Priorities**	**Supporting Quotes from Survey and Interview Data**
1. More flexibility, choice and support for me in school. Not just going back to how it was before COVID-19.	“Give more choice about what we learn. Freedom to choose some online days and some at school days—shorter days.” “For school to be nice to me.”
2. More opportunities for me to make friends, be with friends and have fun.	“More places to go where everyone does what I do so I don’t feel left out.”
3. More support for my emotional and mental health support; no child with SEND should struggle!	“I would make it a law that children like me would be given lots of learning support and emotional support to any child who needed extra help—no child should struggle!”
**Parent/Carer Priorities**	**Supporting Quotes from Survey and Interview Data**
1. More support for my child with SEND’s mental health and emotional wellbeing.	“Her mental health is at an all-time low, she barely leaves her bedroom, she can’t go outside, she sleeps all day and is awake all night.”
2. More support to help me, as a parent, access/receive support for my child with SEND.	“Falling through the system and lack of funding to get him help. We don’t even know what help there is out there as doors have been slammed in our face.”
3. More support for my child to be able to build their social skills, interact with others, and build relationships and friendships.	“The 1000 steps backwards he has taken. We know we have years of work to get him back to the level of social skills and independence we had before the pandemic.”
4. Support to help my child reintegrate back into school and learning following the inaccessible provision during the COVID-19 pandemic.	“Not getting her the right support that she did have before COVID-19 to support her back into full time education. She cannot manage beyond 45 min a day and is doing no work. She isn’t in class and we are having to work hard to get her to even this point.”
5. For my child to have access to appropriate health, social care and education provision for their SEND in a timely and appropriate manner.	“To get the formal test so that she can get the support she needs to make it through her final year.”
6. For my child to have routine, stability, predictability and consistency within their access to education.	“As much consistency as possible. Continued support from the school to enable her learning to continue as uninterrupted as possible.”
7. More accessible activities, youth clubs and holiday clubs.	“SEN friendly places, after school/weekend clubs set up by schools, social interaction sessions as much as possible.”
8. More support and opportunities for my child to gain independence and make up for missed opportunities during the COVID-19 pandemic.	“He is a teenager and has missed a year of developing his independence before leaving school.”

**Table 2:** Most frequent priorities for children and young people with SEND after the COVID-19 pandemic identified by professionals in the surveys and interviews.

**Education Professionals’ Priorities**	**Supporting Quotes from Survey and Interview Data**
1. More funding and focus on mental health and wellbeing in schools, including promoting social and emotional wellbeing and development.	“Additional funding for mental health support which is not available on NHS because they are at capacity.”
2. Increased funding, accessibility, and availability of healthcare services and specialist provision from external agencies, in and out of school.	“Children did not have access to the specialist resources required to address their needs, specific interventions, and resources that some children required e.g., SALT, OT, Physio sessions. Physical resources (chairs, eating equipment, slopes, move and sit cushions) that were not at home.”
3. Improving skills and numbers of staff in mainstream schools—increasing the number of qualified staff, providing SEND-specific training for all staff, increasing the number of teaching assistants (TAs)/1-2-1 support, and providing learning mentors.	“Teacher training no longer prepared teachers for the children’s needs. There are many great resources to support but there is not the time to train properly once in school.”
4. An increased focus on academic ‘catch up’ that is individual to each child (i.e., not compared to peers’ progress), including numeracy and literacy, and targeted interventions/tutoring.	“Funding given to schools to deliver their own catch up. We know what will work. We want to employ staff we know will have an impact—not be directed by the government.”
5. Opportunities for enrichment/extra-curricular activities, including school trips, forest school, access to green spaces and physical exercise.	“Run additional enrichment activities such as school trips as they have missed out on so many experiences and these opportunities also rebuild relationships between peers and staff.”
6. Ensuring appropriate facilities/resources are accessible to all SEND pupils, especially IT/digital infrastructure.	“There needs to be accessible technologies for those with SEND.”
7. “Increase the number of special school places, support special schools to cope with increased capacity, and ensure alternative provision (AP) is always available.	“Improvements to special schools to cope with increase in capacity demands.”
8. Increased funding to support pupils on SEN support and with EHCPs in mainstream schools.	“Continuation of the drive towards inclusion where it is in the best interests of the young person.”
9. Develop and publish expectations for inclusive practice, increase funding to improve inclusion in mainstream schools, and provide staff with training/continued professional development (CPD) in SEND.	“Prioritising the needs of students with SEND not playing lip service to a political agenda.”
10. Increased support with transitions for returning to school.	“Phased return for those that struggle with change and routine.”
11. Allow for a more flexible curriculum that can be reduced and/or tailored to the child.	“Review the curriculum to allow for more flexible teaching which is meaningful to pupils.”
12. Promoting social and emotional development, life skills, and communication development, allowing the option of continued online/blended learning.	“We had to limit our sensory play and cut some on the amount of cooking and life skills we do.”
13. Have clear pathways for progressing EHCPs, introduce strategies to reduce the backlog of EHCP requests and speed up EHCP reviews, and ensure adequate funding is always incorporated into EHCPs.	“Stop fragmenting the services and create faster referral processes.”
14. Reform the EHCP process so that it is the same across all LAs, reduce the appointment backlog, and require LAs to update EHCPs in line with guidance.	“Clear national EHCP funding a postcode lottery at present depending on LA they mean different things in different authorities.”
15. Support for students who have struggled to return to school/have low attendance/have anxiety-related non-attendance (ARNA).	“Their needs have significantly increased especially for SEMH pupils categorised as SEND. Schools need immediate access to funds to meet the demands for 1:1 support so that pupils can quickly and smoothly transition back into school.”
16. Provide support for transition back to school.	“Attendance and preparedness to return to school (due to fears about contact with COVID).”
17. All Government COVID-19 rules with practical advice (e.g., all EHCP students are vulnerable, but it’s not practical for all to attend school at the required social distance).	“We are as usual the hidden group. Government guidance was totally unhelpful and at best tokenistic, contradictory and disconnected from the complexities of running a special school.”
**Health and Social Care Professionals’ Priorities**	**Example supporting Quote from Survey and Interview Data**
1. Improved access for mental health support for children and young people with SEND to mitigate the impact of COVID-19 and lockdowns.	“Young people with SEND have suffered increased anxiety/other mental health issues as a result of isolation and loss of face-to-face youth support during the pandemic.”
2. Improved access to respite and short breaks for children and young people with SEND to mitigate the relentless care provided by families during COVID-19 and lockdowns.	“Increased short break provision generally so that families have enough support to prevent crisis and placement breakdown.”
3. Improved opportunities for social activities and initiatives within the community for children and young people with SEND to reconnect and re-engage after COVID-19.	“Providing school holiday activities to children with SEND and having more accessible and inclusive clubs and activities available.”
4. Children and young people with SEND and families directly informing service design/provision to ensure positive adaptations developed during COVID-19 are retained and prioritised.	“Retainment of online meetings/reviews for those young people and families who benefit from them.”
5. Improved integrated working between health, social care, education and local authorities to ensure that the impact of COVID-19 and lockdowns on children and young people with SEND are considered and addressed using a holistic and person-centred approach.	“Working online makes it easier to attend CPD or multi-agency meetings which may have been hard to do in the past.”
6. Improved equitable access to services/settings for all children and young people with SEND regardless of their SEND, ethnicity, or socio-demographics, to address the additional negative impact of COVID-19 and lockdowns.	“Closing the gaps in access to services for children/YP from BAME and/or lower socio-economic backgrounds.” “Addressing the need of those families who are less digitally literate or do not have the appropriate equipment.”
7. Improved workforce training and development to ensure an adequate workforce is in place to mitigate the impact of COVID-19 and lockdowns.	“A HUGE employment initiative to get staff in & working to a high-quality standard. New staff are not being supported adequately workforce development to upskill and train professionals for preventative work.”
8. Improved access and provision of SEND therapies/services/assessments to meet the needs of children and young people with SEND after the pandemic.	“No redeployment away from vulnerable children. No closing of services or relaxing of statutory requirements.”
9. Improved transitions between services and settings for children and young people with SEND who have had their lives and routines disrupted by the COVID-19 pandemic and lockdowns.	“Transition have been far more challenging as children and young people have not had natural endings, e.g., Leaver’s events, Proms or good physical introductions to new settings which has caused a lot of worry and anxiety.”
**Local Authority (LA) Professionals’ Priorities**	**Key Quotes to Support the Priority Groups from Survey and Interview Data**
1. Improved access to mental health support for children and young people with SEND to mitigate the impact of COVID-19 and lockdowns.	“Increased resources into CAMH and early intervention services for emotional wellbeing services.”
2. Local Authority systems in administering SEND support for children and young people.	“Developing a more effective and agreed inclusion charter and policies within schools.”
3. Improved access/support for families of children and young people with SEND, such as respite and short breaks, to mitigate the relentless care provided by families during COVID-19 and lockdowns.	“Support groups in local family centres, where parents can get together and support one another and not feel alone.”
4. The need for greater investment/funding/resources to support Local Authorities to discharge their duties towards children and young people with SEND.	“Adequate funding to support the number of EHCPs at local authority level.”
5. In-school support: specialist school provision.	“Placement sufficiency—need more special schools.”
6. In-school/college support: Transition post COVID-19/academic progression/attainment.	“More flexible and adaptable system -bespoke arranges that work for individuals—some CYP with SEND has done better with virtual systems we need to understand and incorporate this—blended approach.”
7. In-school support: greater inclusion.	“Focus on inclusion in mainstream schools and how they are supported to support children.” “Ofsted to recognise value of inclusion in their inspections.”
8. Improved access to therapies/support.	“More health provision to make up for the lost therapy.”
9. Broader national recommendations such as changes in law/policy/guidance.	“Tribunal legislation needs revision.” “More legal powers to hold mainstream schools to account to look after children on their roll.”
10. Publication of SEND review (as a national priority).	“Acknowledgement of an already poorly funded system—publication of the SEND Review.”

**Table 3:** Summary of five key priority areas for children and young people with SEND (see Supplementary Materials Table S1 for all priorities and responsibilities).

Priority Area	Evidence from Study	Priorities: Pandemic Management	Priorities: Recovery and Renewal
1. My right to play, socialise, have fun, and be part of my community (UN CRC Articles 15, 23 and 31)	Lack of accessible, adaptable, and available opportunities to join in activities, fulfil sensory/movement needs, engage in specialist play services, and socialise Slower speech and language development, increased isolation/loneliness, and a lack of opportunities to develop independence	Central Government and Local Authorities should ensure: Children with SEND should retain their existing opportunities for play, recreation, and physical activity, facilitated by appropriately trained professionals Children with SEND should be able to play in close pairs/bubbles Opportunities to develop vocational skills should continue	Central Government, Local Authorities, Integrated Care Services, and third sector organisations should ensure: Statutory provision of SEND-accessible and SEND-specific play and recreation areas and services should be ensured, with opportunities developed in collaboration with children with SEND and their families Activities should be regular, ongoing, and facilitated by staff with SEND-specific training SEN/EHCP assessments/annual reviews should consider the need to make play, recreation, and social interventions available
2. My right to support for my social and emotional wellbeing (SEW) and mental health (UN CRC Articles 6 and 24)	Deteriorating mental health, and increased self-harm, anxiety, and behaviours that challenge Difficulties managing change, limitations to exercise, inconsistent guidelines, lack of access to appropriate early interventions, and mental health professionals’ lack of knowledge of SEND	Central Government, Local Authorities, education provision, and national health commissioners/services should ensure: Face-to-face learning and mental health services should continue for children with SEND Tailored public health information/interventions, and school guidance should be provided for and explicitly consider children with SEND Pandemic responses should be underpinned by a Children’s Rights Impact Assessment	Central Government, Local Authorities, Department for Education, Department for Health and Social Care, Public Health England, Health Education England, and education provision should ensure: Initiatives linked to children’s mental health should specifically consider and adapt to the needs of children with SEND All professionals working with or supporting children should receive SEND-specific training, and the workforce in mental health should be increased In line with the NHS Long-Term Plan, children with SEND should be triaged and receive support within four weeks, with alternative SEND-appropriate therapeutic options available
3. My right to flexibility, choice, and support so I can feel safe, belong, and learn in school (UN CRC Articles 28 and 29)	Not all children with SEND were offered access to in-person education, with schoolwork not accessible or appropriately differentiated, resulting in lost learning and disengagement Education staff were redeployed or left their posts Children with SEND faced difficulties with uncertainty and disruptions transitioning back to school Some children with SEND flourished with the increased flexibility to learning	Central Government, Local Authorities, Department for Education, national health commissioners/services, Public Health England, and education provision should ensure: Children with SEND should be offered in-person education Remote learning should be inclusive and appropriately differentiated, with training provided to staff Government guidelines should include a specific focus on children with SEND, which should be clear and timely, with accessible guidelines available for children with SEND and their families Children with SEND should be provided with a tailored transition programme, co-produced with children and families	Central Government, Local Authorities, Department for Education, Ofsted, and education provision should ensure: Inclusive teaching practices should be embedded in schools, with options of a flexible curriculum and opportunities to develop wider skills School staff should be provided with SEND-specific training, and recruitment, training and retention of SEND-related education posts should be prioritised Ofsted should consider the extent to which schools are inclusive and children with SEND feel safe, supported, and included Children with SEND should be offered flexibility for compulsory assessments Schools should implement individual transition plans for children with SEND moving schools
4. My right to health and social care services and therapies in order for me to stay healthy (UN CRC Article 24)	Skilled SEND staff left posts or were redeployed, services stopped or moved online, waiting lists increased, and children with SEND could not access equipment for therapies Children with SEND’s speech and language development deteriorated, many physically deconditioned, and there was a reported increase in safeguarding concerns for children with SEND	Central Government, Local Authorities, Integrated Carer Services, and national health commissioners/services should ensure: Children with SEND should have uninterrupted regular and ongoing access to therapies, in-person if needed Equipment or movement plans should be provided at home Clear and timely guidance from the Government is required, considering the needs of children with SEND Professionals should always be able to see children considered to be at risk face-to-face	Central Government, Local Authorities, Integrated Carer Services, Department of Health and Social Care, and national health commissioners/services should ensure: Reduced waiting times for therapies/treatment/assessment, with streamlined administration processing, and online or in-person options SEND-specific training for all professionals working in health and social care∙ SEN/EHCP assessments/annual reviews to be completed within statutory deadlines
5. My right to support for my parents/carers and my family (UN CRC Articles 18, 27, and 42)	Increased parental stress/burnout and deteriorating mental health and wellbeing No access to carers or respite, increased isolation of families, and difficulty navigating services Increased poverty disproportionately impacted families of children with SEND	Central Government, Local Authorities, education provision, and Department for Education should ensure: All children with SEND to be offered a place in school, with 1-to-1 carers able to attend to children at school/home Children with SEND at home should be provided with necessary equipment to study remotely Respite should continue and parents/carers should be informed of how changes in laws/regulations impact the provision offered to their child	Central Government, Local Authorities, Integrated Care Services, Department of Health and Social Care, and education provision should ensure: Increased provision of parent/carer support groups/opportunities to connect, and support and advocacy services for parents and siblings Meaningful inclusion of parents/carers in SEN/EHCP assessments/annual reviews Clear, accessible and updated Local Offer Streamlined processes for applying for support/services Access to free training for parents/carers to improve SEND health literacy and knowledge of children’s rights

## Data Availability

The data that support the findings of this study are available on request from the corresponding author. The data are not publicly available due to privacy or ethical restrictions.

## Supplementary Materials

The following supporting information can be downloaded at: https://www.mdpi.com/article/10.3390/children12070827/s1, Figure S1: Five themes identified from Phases 1 and 2 for presentation in Phase 3 workshop; Table S1: Full list of priorities for policy and practice .
