# Supplementary material for: Rights-Based Priorities for Children with SEND in the Post-COVID-19 Era: A Multi-Method, Multi-Phased, Multi-Stakeholder Consensus Approach
Source: Children (Basel). 2025 Jun 23;12(7):827. doi: 10.3390/children12070827 (PMC12293363; doi:10.3390/children12070827)
Supplement: Supplementary file 1 [file children-12-00827-s001.zip › children-3671602-supplementary.pdf]

Figure S1. Five themes identifed from Phases 1 and 2 for presentation in Phase 3 workshop

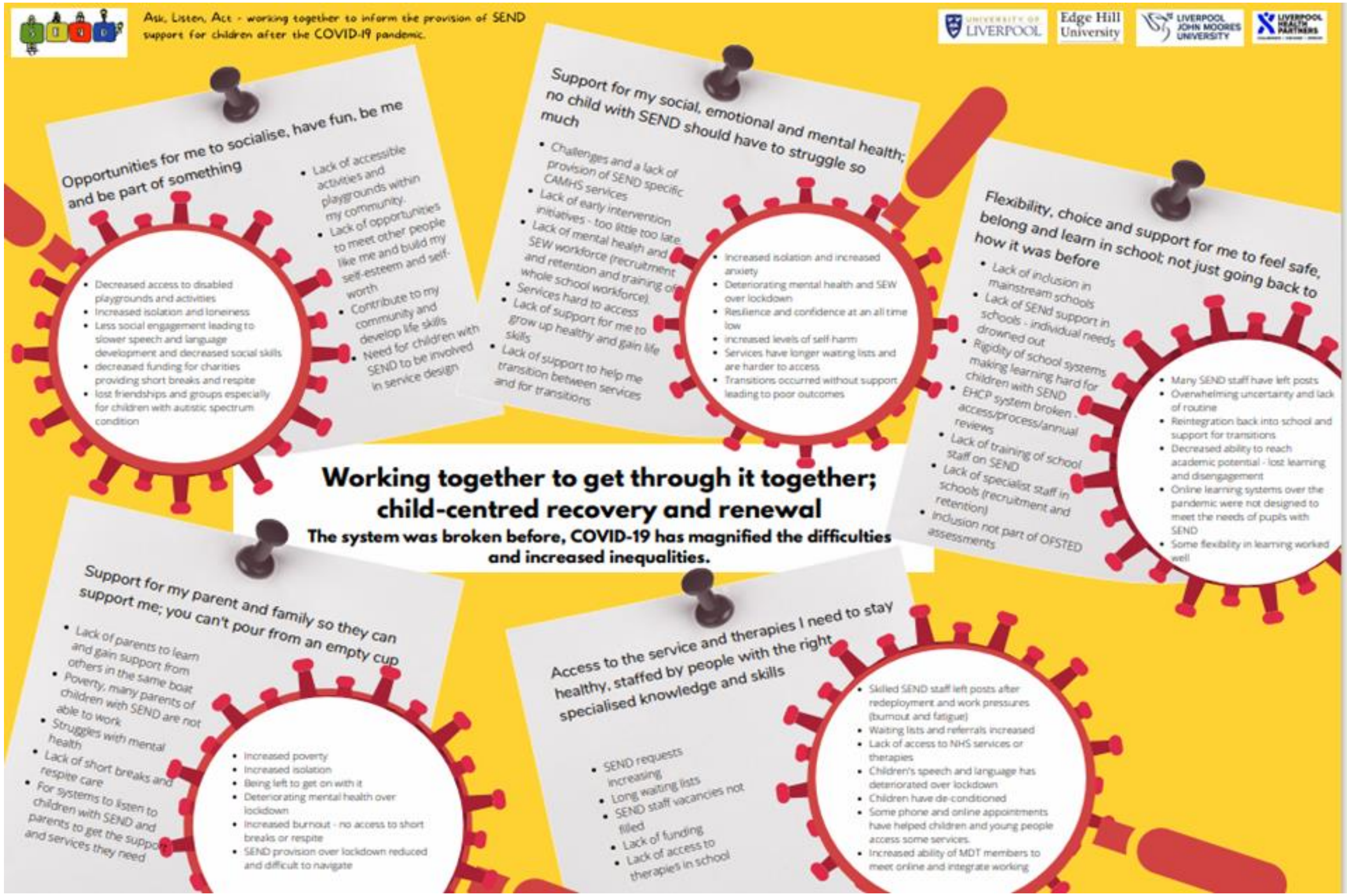

Table S1. Full list of priorities for policy and practice

My Right to Play, Socialise, Have Fun, and be Part of my Community

Article 31, -Every child has the right to relax, play and take part in a wide range of cultural and artistic activities.
Article 15 - Every child has the right to meet with other children and to join groups and organisations, as long as this does not stop other people from enjoying their rights.
Article 23 - A child with a disability has the right to live a full and decent life with dignity and, as far as possible, independence and to play an active part in the community. Governments must do all they can to support disabled children and their families.

Evidence from the study	Policy and practice priorities specific to pandemic management	Policy and practice priorities linked to recovery and renewal
• A lack of access to opportunities for children and young people to fulfil sensory (vestibular) needs for movement during the pandemic<br><br>• A lack of accessible, adaptable and available specialist play services (disabled playgrounds were locked and specialist play providers closed)<br><br>• A lack of access to accessible and adaptable opportunities to be active and join in activities<br><br>• Lost friendships and social connections as a result of the pandemic, especially for children and young people with autistic spectrum condition (ASC)	• Children and young people with SEND should retain existing opportunities for play and physical activity, particularly where lack of it may compound and exacerbate existing SEND (e.g. the need for children with ADHD/ADD to move, sensory circuits for those with Sensory Processing Difficulties, or maintaining a routine for children with ASC).<br><br>• Play and activity sessions during lockdown should be facilitated by credible and appropriately SEND trained professionals who can provide 1-2-1 support.<br><br>• Outdoor playgrounds and sensory rooms should remain open for children and young people with SEND and there should be greater coordination between Local Authorities and Central Government regarding funding issues to ensure playgrounds are accessible, adaptable and available.<br><br>• Areas for play and recreation should be provided within schools that in previous lockdowns were closed (e.g hydrotherapy pools and sensory rooms should be maintained and kept open as essential for children and young people with SEND).	• Local Authorities should ensure statutory provision of SEND-accessible play and recreation services.<br><br>• Local Authorities, Integrated Care Services, and Third Sector Organisations should design and deliver play and recreation activities in collaboration with children and young people with SEND and their families.<br><br>• Any new centrally-funded recovery programmes and initiatives (e.g. holiday activities and food programmes) need to include activities that are accessible and adaptable to children and young people with SEND.<br><br>• Local Authorities need to provide SEND-specific play and recreation activities and groups, which are accessible, adaptable and available and designed to meet the needs of children and young people with all types of SEND.

My Right to Play, Socialise, Have Fun, and be Part of my Community

Evidence from the study	Policy and practice priorities specific to pandemic management	Policy and practice priorities linked to recovery and renewal
• A lack of access to reasonably adjusted opportunities to socialise, resulting in decreased social skills and increased isolation and loneliness<br><br>• Fewer opportunities for social engagement leading to slower speech and language development.<br><br>• A lack of opportunities to build important life skills and independence within the community that were tailored to children and young people's needs.<br><br>• Some charitable services which are important for children and young people with SEND were at a real risk of closing down e.g. horse riding and sensory rooms	• Government guidelines should allow children and young people with SEND to play in close pairs or social bubbles and engage in repeated mixing, and Local Authorities should facilitate activities outside.<br><br>• Older children and young people with SEND should have independence and life skills built into their curriculum and this should remain accessible/ongoing if there are any further lockdowns.<br><br>• Vocational skills (e.g. work experience) should continue where possible.	• Local Authorities and central Government should ensure non-SEND specific play and recreational activities are inclusive and accessible for children and young people with SEND.<br><br>• Local Authorities and central Government should ensure that activities should be regular and ongoing (not long waiting lists, not limited to 6 sessions).<br><br>• Local Authorities and central Government should ensure that activities are facilitated by credible staff who have been provided with SEND-specific training and who can provide 1-2-1 support.<br><br>• Local Authorities need to work with schools to promote and deliver accessible and adaptable play and recreational activities.<br><br>• As part of EHCP assessments/annual reviews, Local Authorities should specifically consider the need to make individual play and recreational social communication interventions available for children and young people with SEND.

My Right to Support for my Social and Emotional Wellbeing (SEW) and Mental Health

Article 6 Every child has the right to life, survival and development. Governments must do all they can to ensure that children survive and develop to their full potential.
Article 24 Every child has the right to the best possible health. Governments must provide good quality health care, clean water, nutritious food, and a clean environment and education on health and well-being so that children can stay healthy.

Evidence from our study	Policy and practice priorities specific to pandemic management	Policy and practice priorities linked to recovery and renewal
• Children and young people with SEND experienced deteriorating mental health and social-emotional wellbeing (SEW) over lockdown.<br>• Children and young people with ASC struggled with a change to routine (anxiety, uncertainty, behaviours that challenge).<br>• Children and young people with ADHD/ADD struggled with distress and demonstrated increased behaviours that challenge due to limitations to exercise and activity.<br>• Education professionals reported seeing increased levels of self-harm in pupils with SEND.	• Children with SEND should be offered the option of face-to-face in-person learning in school throughout any future lockdowns.<br>• NHS England and Local Authorities should provide tailored information and interventions to provide public health information to children and young people with SEND (e.g. social stories to explain change and COVID-19 restrictions).<br>• Children with SEND should always have their right to play and engage in leisure activities upheld in future lockdowns.<br>• If there is another lockdown, there needs to be clear legislative guidance for Local Authorities, schools, and parents from the outset, provided in a timely manner, which explicitly considers children with SEND. This needs to be clearly communicated to children to decrease uncertainty and allow for preparation for new change (e.g. via social stories etc.) .	• Department for Education should require all schools to deliver universal, mental health prevention and promotion programmes for all children and young people with SEND (e.g. teaching healthy coping strategies, mental health literacy).<br>• Central Government, Department of Health and Social Care, Department for Education, and Public Health England initiatives linked to children and young peoples' mental health (e.g. mental health support teams, psychological first aid training courses, and mental health and wellbeing recovery action plan group) need to consider and specifically address the mental health and wellbeing needs of children with SEND.<br>• SEW and mental health is the responsibility of all school staff and the Department for Education/Local Authorities should ensure specific SEND training is provided for all (during core teacher training and post qualification), to ensure early intervention is provided.

My Right to Support for my Social and Emotional Wellbeing (SEW) and Mental Health

Evidence from our study	Policy and practice priorities specific to pandemic management	Policy and practice priorities linked to recovery and renewal
• Children and young people with SEND experienced worsening mental health and anxiety over leaving the house.<br><br>• There were delays in children and young people with SEND accessing appropriate early interventions for their SEW and mental health.<br><br>• Mental health practitioners do not have adequate knowledge of SEND specific mental health needs (e.g. ASC).<br><br>• The delivery of SEW and mental health services were not tailored to meet the SEND specific needs of these children and young people.<br><br>• Governments guidance was inconsistent and rapidly changing on how mental health service provision should be delivered.<br><br>• Mental health services for children and young people with SEND have longer waiting lists and are harder to access.	• On the return from any further restrictions which impact children with SEND, or any school closures/long term absences, there needs to be an individually tailored transition plan for children with SEND from being at home to going to school/clubs/being outside with other people, to ensure a smooth transition (this could include a revisiting of an EHCP for those children who have one).<br><br>• School staff should check in with parents/carers/children regularly (e.g. minimum once per week) to identify any developing SEW or mental health issues, and then offer appropriate support.<br><br>• SEW and mental health services should continue to offer the option of face-to-face appointments, with necessary Personal Protective Equipment (PPE), for those children and young people with SEND who struggle to communicate via video/telephone.<br><br>• Any Government or Local Authority responses to the pandemic, including future restrictions or lockdowns, need to consider evidence which demonstrates how lockdowns impact on the mental health of children with SEND, and ensure provision is put in place to mitigate these. This should be underpinned by a Children's Rights Impact assessment (CRIA).	• Health Education England should ensure that GPs and primary care services should receive training in appropriate services for diagnosis and support for children with SEND experiencing mental health difficulties.<br><br>• Department for Education, Department of Health and Social Care and Health Education England should ensure Investment in training positions to increase the workforce within SEW and mental health, both in and outside of school (e.g. CYP Psychological Wellbeing Practitioner, Education Mental Health Practitioner). Training in these positions should include a SEND-specific component.<br><br>• Local Authorities should ensure there is a comprehensive and up-to-date access point as part of the Local Offer about the statutory and voluntary services for SEW and mental health which are available for children with SEND.<br><br>• In line with the NHS long-term plan, all children with SEND should be triaged and begin receiving support for mental health difficulties within 4 weeks of referral, based on their individual specific needs.

My Right to Support for my Social and Emotional Wellbeing (SEW) and Mental Health

Evidence from our study	Policy and practice priorities specific to pandemic management	Policy and practice priorities linked to recovery and renewal
	• Mental health services need to maintain the same, or increased, level of service for children with SEND if lockdown restrictions come back into force.	• Department of Health and Social Care/The Health and Care Professionals Council should provide/require specific SEND training (both core training and post-qualification) for all mental health professionals working with children and young people (e.g. clinical psychologists, psychological wellbeing practitioners, school counsellors).<br><br>• Department for Education/Local Authorities should provide all professionals (school nurses, teachers, teaching assistants) who have contact with children and young people with SEND with specific SEND training.<br><br>• Department of Health and Social Care and Integrated Care Systems should ensure alternative, SEND-appropriate, therapeutic options are readily available and offered to children and young people with SEND (e.g. music therapy)

My right to flexibility, choice, and support so I can feel safe, belong, and learn in school

Article 28 (right to education) Every child has the right to an education. Primary education must be free and different forms of secondary education must be available to every child. Discipline in schools must respect children's dignity and their rights.
Article 29 (goals of education) Education must develop every child's personality, talents and abilities to the full. It must encourage the child's respect for human rights, as well as respect for their parents, their own and other cultures, and the environment

Evidence from our study	Policy and practice priorities specific to pandemic management	Policy and practice priorities linked to recovery and renewal
• Not all children and young people with SEND (including some with EHCPs) were offered access to in person education.<br><br>• Not all EHCP assessments and reviews were conducted on time.<br><br>• Many children and young people with SEND's needs according to their EHCP were not met over the pandemic.<br><br>• Government advice and guidance for education settings was unclear and delivered too late to operationalise safely.<br><br>• Education staff were re-deployed away from being able to deliver SEND support.	• Department for Education/Local Authorities should ensure all children with SEND (whether they have an EHCP or not) are offered the opportunity to attend in person education provision full time where possible.<br><br>• Where children with SEND must learn remotely, schools (with support from the Local Authority/Department for Education) should ensure online learning is inclusive and appropriately differentiated.<br><br>• Government guidance for COVID-related restrictions should include a specific focus on and prioritised delivery of education and learning for children with SEND.<br><br>• Local Authorities should ensure EHCP assessments and annual reviews continue to be completed within the statutory deadlines, with the option of face to face or online meetings offered to families.	• Department for Education should require all mainstream schools to embed inclusive teaching practises in the classroom (e.g. active listening, visual aids and auditory memory techniques explicitly taught and built into daily classroom life).<br><br>• Department for Education should allow schools the option of a flexible curriculum (e.g. subjects) and delivery (e.g. outdoor learning) that supports children with SEND (e.g. opportunities for blended learning online and in school), and they should provide schools with the resources and training to do this.<br><br>• Local Authorities should ensure EHCPs are specified and quantifiable as per the Children and Families Act 2014.<br><br>• Department for Education should provide schools with the opportunity to promote wider skills for children with SEND (e.g. life skills, transitions to adulthood, work experience).

My right to flexibility, choice, and support so I can feel safe, belong, and learn in school

Evidence from our study	Policy and practice priorities specific to pandemic management	Policy and practice priorities linked to recovery and renewal
• Many education staff who provide SEND support have left their posts during the pandemic.<br><br>• Children and young people with autistic spectrum condition (ASC) experienced anxiety and stress as a result of the uncertainty and lack of routine around education and learning.<br><br>• There was a lack of support for children and young people with SEND to reintegrate back into school, and a lack of support for key educational transitions caused high levels of anxiety and disengagement from the learning environment.<br><br>• Children and young people with SEND experienced lost learning and increased disengagement over the pandemic, resulting in a decreased ability to reach their academic potential.	• Schools (with support from the Local Authority/Department for Education) should ensure a child's educational provision detailed within their EHCP/Individual Education Plan/Early Help Assessment Tool is still delivered, even during periods of lockdown or pandemic restrictions.<br><br>• Clear and timely guidance and information from the Government needs to be provided to Local Authorities and schools regarding shielding and restrictions, to allow enough time for professionals to plan appropriate support and access for children with SEND.<br><br>• Department for Education, NHS England and Public Health England should provide children with SEND with clear and accessible tailored information on any updated COVID guidance to help reduce anxiety.<br><br>• Schools (with support from the Local Authority/Department for Education) need to offer children and young people with SEND an individually tailored transition programme, co-produced with children and parents, to facilitate their return back to school after any lockdown/periods of time in home-schooling (e.g. virtual transition meetings, social communication sessions and social stories to support transitions).	• Department for Education should increase the links for education staff between special schools and mainstream schools (e.g. school buddy system), to provide opportunities for shared learning, training, and the sharing of resources.<br><br>• Department for Education should ensure all school staff receive SEND-specific training (both core training and post-qualification), to effectively support children and young people with SEND.<br><br>• Ofsted criteria should incorporate the extent to which schools are inclusive and children with SEND feel psychologically and physically safe, supported, and included.<br><br>• Department for Education should invest in recruitment, training, and retention of SEND-related education posts (e.g. Learning Support Assistants, SENCO's and SEND support staff), e.g. offering bursaries to enter the profession, increasing remuneration in recognition of the skills and challenges such positions may entail.

My right to flexibility, choice, and support so I can feel safe, belong, and learn in school

Evidence from our study	Policy and practice priorities specific to pandemic management	Policy and practice priorities linked to recovery and renewal
• Many parents struggled to support their child's additional learning needs during homeschooling.<br><br>• There was reduced communication between parents and education providers and the Local Authority about their child's learning needs and wellbeing.<br><br>• Families had a lack of access to appropriate learning equipment or technology to meet their child's needs.<br><br>• Online learning systems used over the pandemic were not designed for and did not meet the additional learning needs of pupils with SEND.<br><br>• Some children and young people with SEND flourished with an increased flexibility to learning and children and families expressed a wish for this flexibility to be maintained.	• Local Authorities should ensure children and young people with SEND moving educational institutions (e.g, starting school, year 6 pupils or year 11 pupils) are offered the opportunity to visit new schools/education providers prior to moving and continue with transition plans despite COVID restrictions. They should support schools to facilitate this.<br><br>• Department for Education should provide education staff with training on how to provide and adapt online learning for children with SEND, to help deliver effective education in future lockdowns or periods of time when children with SEND need to isolate.<br><br>• Schools (with support from the Local Authority/Department for Education) should ensure the focus of any recovery curriculum is on mental health and wellbeing, in addition to making up for missed education components.<br><br>• Schools (with support from the Local Authority/Department for Education) should limit the amount of new pandemic-related responsibility placed on staff, provide guidance on ensuring their safety in school, and offer effective wellbeing support	• Local Authorities should ensure that schools nationally implement individual transitioning plans to support all children with SEND (not just those with EHCPs) who are moving schools, and enable a visit to a new school.<br><br>• Department for Education should avoid pressure on academic 'catch-up'. The Government guidelines should include recommendations for supporting children with SEND during this time.<br><br>• Schools should restore or establish means of day-to-day communication with parents/carers to pre-pandemic methods, to facilitate communication and ensure that a child's daily needs are known by educational professionals.<br><br>• Department for Education should offer flexibility for compulsory assessments (e.g. GCSEs) for children and young people with SEND to be assessed in a way that best suits their needs (e.g. exams or coursework).

My Right to Health and Social Care Services and Therapies in Order for me to Stay Healthy

Article 24 Every child has the right to the best possible health. Governments must provide good quality health care, clean water, nutritious food, and a clean environment and education on health and well-being so that children can stay healthy.

Evidence from our study	Policy and practice priorities specific to pandemic management	Policy and practice priorities linked to recovery and renewal
• Skilled SEND staff in health and social care left posts after redeployment and work pressures (burnout and fatigue).<br><br>• Waiting lists and referrals for access to health care services and therapies increased for children and young people with SEND.<br><br>• There was a lack of access to NHS services or therapies for children and young people with SEND during the pandemic - many services 'just stopped'.<br><br>• Access to NHS services or therapies was only available by video or phone which was not suitable to many children and young peoples' needs.<br><br>• Some phone and/or online appointments and multi-disciplinary team meetings have helped parents and children access some health and social care services	• Integrated Care Services for health and social care should ensure uninterrupted regular and ongoing access to therapies (especially in school) for children and young people with SEND, even during lockdowns and restrictions.<br><br>• NHS England should ensure families are offered face-to-face access to therapies and health services for children and young people with SEND.<br><br>• Local Authorities should ensure sensory circuits/movement plans put in place by Occupational Therapists are maintained in school settings and where not possible, shared with parents/carers for a child to do at home.<br><br>• Clear and timely guidance and information from the government is needed regarding shielding and restrictions, to enable professionals adequate time to plan appropriate support and access to children with SEND.	• Investment and resources (workforce, funding, equipment, facilities) are needed from Department of Health and Social Care/NHS England to reduce waiting lists for therapies/treatment and health assessments. This would be evidenced by quarterly updates.<br><br>• Local Authorities should ensure that EHCP assessments and annual reviews are completed within the statutory deadlines, with families being offered face-to-face or online meeting options.<br><br>• NHS England/Integrated Care Services should provide options for face-to-face or online therapies and assessments based on families' preferences.<br><br>• NHS England/Integrated Care Services should streamline the administration process for accessing health and social care services and therapies, reducing the volume and length of forms and phone calls needed.

My Right to Health and Social Care Services and Therapies in Order for me to Stay Healthy

Evidence from our study	Policy and practice priorities specific to pandemic management	Policy and practice priorities linked to recovery and renewal
• A lack of access to equipment for therapies was experienced by many children and young people with SEND (delays to access and difficulty installing equipment at home).<br><br>• Children and young peoples' speech and language development deteriorated over lockdown.<br><br>• Many children and young people with SEND have physically deconditioned.<br><br>• The increased use of online platforms has led to the increased ability of multi-disciplinary team members to meet and integrate working practises.<br><br>• There was a reported increase in safeguarding concerns for children and young people with SEND.	• Local Authorities should ensure that EHCP assessments and annual reviews are completed within the statutory deadlines with families being offered face-to-face or online meeting options, regardless of COVID restrictions or future lockdown.<br><br>• Integrated Care Services should provide tailored physical activities for children with SEND to improve and maintain their physical health, regardless of future COVID restrictions or lockdowns (e.g. hydrotherapy, occupational therapy).<br><br>• Where there are safeguarding concerns, Local Authorities should ensure health and social care professionals can always see children considered to be at risk face-to-face throughout lockdowns with suitable PPE.	• Department of Health and Social Care (Health Education England) should ensure all professionals working in health and social care are trained (both core training and post-qualification) in and are knowledgeable on SEND-specific conditions and the physical and mental health implications of these.<br><br>• NHS England/Integrated Care Services should ensure that first assessment occurs within 3 months and the pathway for ASC and ADHD/ADD to diagnosis should be no longer than 12 months.

My Right to Support for my Parents/Carers and my Family

Article 18 - Both parents share responsibility for bringing up their child and should always consider what is best for the child. Governments must support parents by creating support services for children and giving parents the help they need to raise their children.
Article 27 - Every child has the right to a standard of living that is good enough to meet their physical and social needs and support their development. Governments must help families who cannot afford to provide this
Article 42 (knowledge of rights) Governments must actively work to make sure children and adults know about the Convention.

Evidence from our study	Policy and practice priorities specific to pandemic management	Policy and practice priorities linked to recovery and renewal
• Increased poverty and deprivation over the pandemic has disproportionately impacted families of children and young people with SEND.<br><br>• Increased isolation of families, and families being 'left to get on with it'.<br><br>• Deteriorating mental health and general wellbeing of parents of children and young people with SEND over the pandemic.<br><br>• Increased parental stress and burnout due to no access to carers, short breaks, or respite.	• All children and young people with SEND (not just those with EHCPs) need to be offered a place in school to enable working parents to continue working, should there be further lockdown restrictions.<br><br>• Government restrictions should always allow any 1-to-1 carers to attend to children and young people with SEND in their home and in school, with appropriate PPE.<br><br>• Schools should ensure that children and young people with SEND in receipt of free school meals have money vouchers sent home if further lockdown measures mean they are not in school.	• Local Authorities should increase the provision of parent support groups and schools should provide opportunities for parents to connect (this may be particularly important for parents of children in specialist provision).<br><br>• Department of Health and Social Care should increase resources and enhance support for statutory, charitable and centrally-funded organisations to provide support and advocacy services for parents/carers and siblings of children with SEND.<br><br>• Local Authorities and schools should acknowledge parent/carers' expertise and include them meaningfully in meetings, EHCP assessments and Annual Review meetings.<br><br>• Local Authorities should update the Local Offer to include clear and accessible information for parents about their child's legal entitlements.

My Right to Support for my Parents/Carers and my Family

Evidence from our study	Policy and practice priorities specific to pandemic management	Policy and practice priorities linked to recovery and renewal
• Parents found access to SEND provision over lockdown reduced and difficult to navigate.	• Department for Education should ensure necessary ring-fenced funding is put in place for schools to be able to provide equipment needed for children and young people with SEND to facilitate their ability to study remotely. This should go beyond the provision of laptops/tablets (e.g. ear defenders, weighted items, fidget items, theraputty, adaptive pens, seating adaptations, overlays etc.).<br><br>• Government guidance should ensure that respite and short break provision for children and young people with SEND can be maintained in any future lockdowns.<br><br>• Local Authorities should update the Local Offer to include information about changes to service access and provision during lockdowns and restrictions.<br><br>• Local Authorities should inform parents/carers of children and young people with an EHCP of any change in laws/regulations/restrictions and explain specifically how it will impact the provision offered to their child, and what support remains available to them during any restrictions. They should also facilitate navigation of SEND support and provision available.	• Local Authorities should update the Local Offer to include clear and accessible information for parents about available services for their child with SEND.<br><br>• Integrated Care Services should streamline the processes for applying to Disability Living Allowance, schools and other services, so the same forms do not need to be completed multiple times.<br><br>• Department of Health and Social Care should provide parents/carers with access to training from statutory, charitable, and centrally-funded organisations to improve their SEND health literacy and knowledge of children’s rights.
